# The role of surgical margins in atypical Lipomatous Tumours of the extremities

**DOI:** 10.1186/s12891-018-2053-3

**Published:** 2018-05-17

**Authors:** Jessica Rauh, Alexander Klein, Andrea Baur-Melnyk, Thomas Knösel, Lars Lindner, Falk Roeder, Volkmar Jansson, Hans Roland Dürr

**Affiliations:** 1 Musculoskeletal Oncology, Department of Orthopaedic Surgery, Physical Medicine and Rehabilitation, University Hospital, Ludwig-Maximilians-University, Campus Grosshadern, Marchioninistr. 15, D-81377 Munich, Germany; 2 Institute of Radiology, University Hospital, LMU, Munich, Germany; 3 Institute of Pathology, University Hospital, LMU, Munich, Germany; 4 Department of Medicine III, University Hospital, LMU, Munich, Germany; 5 Department of Radiotherapy, University Hospital, LMU, Munich, Germany; 6 CCU Radiation Oncology, German Cancer Research Center (DKFZ), Heidelberg, Germany

**Keywords:** Atypical lipoma, Surgery, Recurrence, Dedifferentiation, Prognostic factors

## Abstract

**Background:**

Atypical lipomatous tumours (ALT) are common adipocytic tumours. Due to their large size and deep-seated location, wide resection might result in severe functional deficits. The question which margins should be aimed is hence discussed controversially.

**Methods:**

Forty consecutive patients underwent limb-sparing resections. Margins were defined as R0 (wide resection), R1 (marginal resection) or R2 if tumour was left. All patients were followed for evidence of local recurrence or remote metastases. Overall and recurrence-free survival was calculated.

**Results:**

The mean age at the time of surgery was 61.9 years. The mean tumour diameter was 17 cm with no patient having metastatic disease. In 8 cases a wide (R0) resection, in 31 cases a marginal (R1) and in one patient a R2-resection was performed. The median follow-up time was 40 months. Four patients died due to causes that were not tumour-related. 3 (7.5%) patients (all R1) developed local recurrences. Two of our 3 recurrences in this series occurred in 6 already recurring cases. We observed no dedifferentiation of tumours and no metastatic disease.

**Conclusions:**

ALT represents a comparatively common diagnosis in large deep-seated lesions of the extremities, especially in patients over 60 years. Marginal resection shows an acceptable rate of local recurrence. The risk of dedifferentiation as proven also in a metaanalysis of the English literature of the last 30 years is close to 1%, metastatic disease is exceedingly rare.

## Background

Well into the 1970s, the term “well-differentiated liposarcomas” was used to describe a class of adipocytic soft tissue tumours with local aggressive behavior but typically without metastatic spread. Based on this particular behavior, they have been renamed as “atypical lipomatous tumours (ALT)” or “atypical lipomas” if seen in the extremities or at the trunk where complete surgical excision is easier achievable than in a retroperitoneal location [1, 2]. In body regions that are more difficult to access surgically and where local recurrence is common and where a lethal outcome is possible without dedifferentiation of the tumour or metastatic disease, the term “well-differentiated liposarcoma (WDLS)” is still more appropriate [3, 4]. ALTs are with a frequency of 40–45% the most common adipocytic tumours, often seen after the fifth decade of life with a slight male predominance [3, 5]. Growing slowly this may result in comparatively large tumours.

On the benign side of the spectrum, large deep-seated lipomas do not show an overexpression of MDM2 and CDK4, thus allowing for a clear histopathological distinction from more aggressive lesions.

So the decision whether to classify a histolopathologically well-differentiated liposarcoma as an ALT or as a WDLS is mainly based on tumour location and surgical resectability and reflects the course of the disease with respect to the incidence of dedifferentiation and distant metastases [6].

Based on their typically large size and deep-seated location, a wide resection might result in severe functional deficits. So a controversial discussion about what type of margins (marginal vs wide resection) should be aimed for and whether adjuvant radiotherapy might reduce the risk of local recurrence is still ongoing [7]. This study reflects the experience of treating these lesions at a referral sarcoma center.

## Methods

From 1988 through 2015, 40 consecutive patients with ALT of the extremities and the trunk were treated at our institution, 39 of them after 2002. All tumours were located deep to the fascia and had a diagnosis of ALT based on histopathological features and immunohistochemistry.

In terms of preoperative imaging, predominantly magnetic resonance imaging (MRI) and in some cases computed tomography (CT) was used to define size and precise location of the tumour. A CT scan of the chest was the standard study to exclude metastatic disease.

All patients underwent limb-sparing surgical resection. The margin was defined as R0 if a rim of sound tissue around the lesion was present (wide resection) or R1 if the margins were contaminated but the tumour capsule with the latter remaining closed (marginal resection). In few selected patients, part of the tumour was left as part of the surgical strategy and these were classified as a R2 resection.

**Fig. 1:**
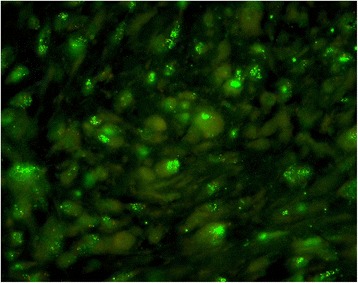
Fluorescent in situ hybridization (FISH) analysis with MDM2 amplification with clusters of green signals. Centromer is red

In all cases, we performed an MDM2 and CDK4 immunostaining as surrogate marker for MDM2 gene amplification. In ambivalent cases MDM2 fluorescent in situ hybridization (FISH) analysis was performed (Fig. 1).

All patients were followed for evidence of local recurrence or distant metastases in general by MRI scans and chest x-rays.

For statistical analysis, overall and recurrence-free survival were calculated according to the Kaplan-Meier method. Significance analysis was performed using the Log-Rank Test or the Chi-Square Test. The data analysis software used was MedCalc®.

## Results

### This series

The mean age of the 21 male and 19 female patients was 61.9 years (range: 9–86). The lower extremity was involved in 33 cases (29 thigh, 4 lower calf), the upper and lower arm in 1 each, the axilla in 2 and the trunk in 3 patients. The mean tumour size was 17 cm (range: 4–65).

The mean duration of symptoms prior to surgery was 26 months (range, 1–323): 38 (95%) patients complained of swelling, 11 (28%) of pain. Neurological impairment (sensory) or restriction of movement was seen occasionally. Two patients were diagnosed as a consequence of ruling out a suspected deep vein thrombosis. Thirty-one patients had a biopsy taken at our institution or existing histopathology studies from previous surgeries. Local recurrence after surgery at other institutions was seen in 6 cases and occurred at a mean of 15 months after the preceeding surgery. No patient had evidence of metastatic disease.

**Fig. 2:**
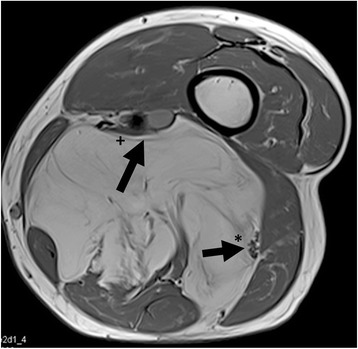
A T2-weighted MRI scan shows an atypical lipoma in the dorsal aspect of the thigh in an 81-year old patient. The sciatic nerve (*) and the major vessels (+) are close to the tumour

**Fig. 3:**
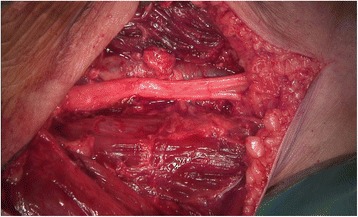
Same patient as Fig. 2. The tumour has been marginal resected keeping the sciatic nerve and the major vessels

In 8 cases a wide (R0) resection, in 31 a marginal (R1, Figs. 2 and 3) and in one patient with recurrent disease after 5 previous surgeries (71 years old, involvement of the sciatic nerve) a R2-resection was performed. Surgical complications included transient motor deficits in 3 patients, prolonged wound healing in 3, hematoma in 3, one infection and lymphedema in one patient. In 4 patients an adjuvant radiotherapy was performed. Two of these patients suffered from recurrent lesions and two from primary disease with infiltration of critical structures and marginal resections.

The median follow-up time was 40 months (range, 2–151). Nine patients had a follow-up of less than 9 months whereas 13 had a follow-up of more than 60 months. Four patients died due to non-tumour-related causes.

**Fig. 4:**
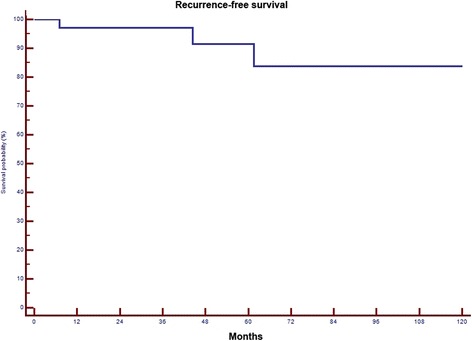
Local recurrence-free survival in 40 patients with ALS

**Fig. 5:**
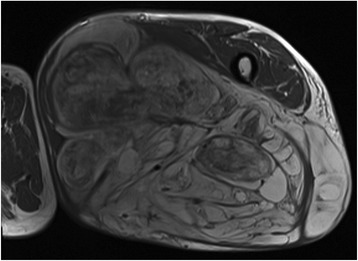
A T2-weighted MRI scan shows an atypical lipoma in the dorsal aspect of the thigh in an 85-year old patient. This was the 13th local recurrence of the tumour classified always as “lipoma”

**Fig. 6:**
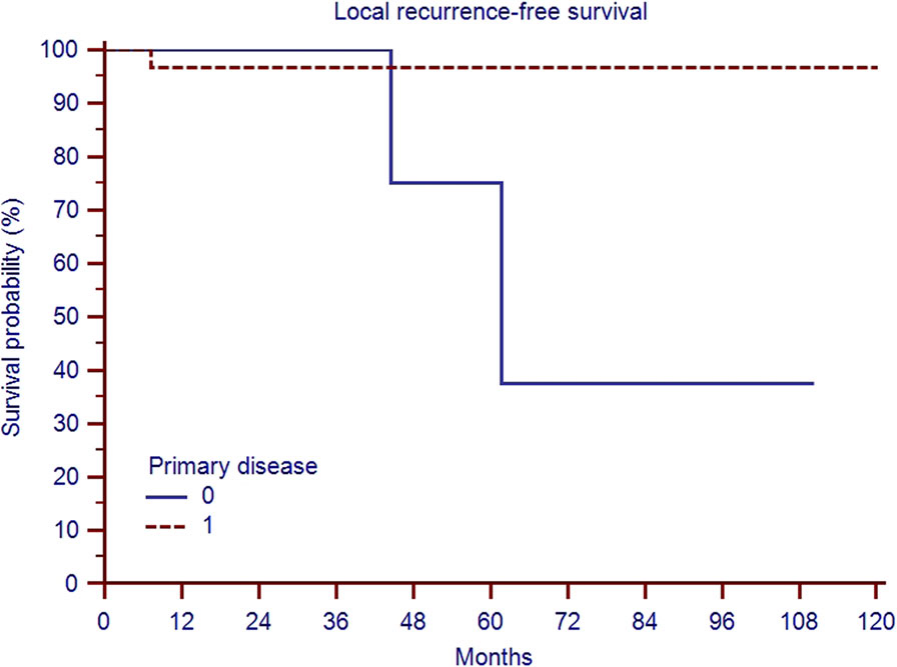
Local recurrence-free survival in 34 patients with primary ALS and 6 patients with recurrent disease (*p* = 0,0285)

The 5-year local recurrence free survival in this cohort was 95%. In total, 3 (7.5%) patients developed local recurrences at 7, 44 and 62 months after surgery, respectively (Fig. 4). All three patients had a marginal (R1) resection (n.s.). One of the patients had re-resection and is currently tumour-free 9 years after the second resection. Another patient has a small recurrence (after his 4th surgery) without any symptoms and is under “watchful waiting” 6 years after surgery. The third, an 89-year old patient with 14 prior surgeries and with severe heart disease has mild symptoms and has elected not to undergo further surgery (Fig. 5). We observed no case of dedifferentiation and no metastatic disease during their follow-up. In comparison to the rest of the cohort, these three patients had larger tumours (mean 26 compared to 16 cm, n.s.). Two of the 3 recurrences occurred in 6 already recurrent cases and only one after the 34 primary resections (Fig. 6, *p* = 0,0285). Out of the 4 irradiated cases none developed local recurrence (n.s.).

### Literature

In addition, the English literature of the last 30 years including series with more than 10 cases was reviewed in detail. The results are summarized in Table 1. In total, 1143 patients are described in the papers reviewed. In 1043 of these patients, the margin status was mentioned. 59% of these cases had a marginal and 41% had a wide resection. Regarding local recurrence, information could be extracted in 701 of the patients with defined margins. Local recurrence developed in 17% of the marginally resected and in 7% of the wide resected patients (*p* < 0.05). In the whole group of 1143 patients, local recurrence was seen in 174 (15%) cases. Of these patients 14 (8%) developed a dedifferentiation in the context of local recurrence. No patient in the whole group was shown to have developed metastatic disease. The mean time until recurrence was 5.5 years and in 6 of 10 studies the mean time until local relapse was greater than 5 years.

**Table 1:** Summary of oncologic outcome in published series of ALT of extremities and trunk wall.

			Local Recurrence			Dedifferentiation of recurrences	Metastatic disease	Mean time to recurrence (years)
Author	Year	Patients (n)	Wide Resection	Marginal Resection	Total			
Azumi et al. [20]	1987	24			7/24 29%	0/7 0%	0	
Weiss et al. [6]	1992	46			20/46 43%	3/20 15%	0	
Lucas et al. [25]	1993	32	1/9 11%	14/23 61%	15/32 47%	1/15 7%	0	7
Rozental et al. [28]	2002	31	1/9 11%	15/22 68%	16/31 52%	4/16 25%	0	4,7
Kooby et al. [24]	2003	91	0/28 0%	5/63 8%	5/91 5%	3/5 60%	0	6,8
Bassett et al. [23]	2004	51	0/0	14/51 27%	14/51 27%	1/14 7%	0	4
Sommerville et al. [22]	2005	61	0/0	5/61 8%	5/61 8%	0/5 0%	0	3,2
Evans et al. [21]	2007	11			1/11 9%	0/1 0%	0	
Zagars et al. [31]	2007	15			8/15 53%	0/8 0%	0	
Billing et al. [5]	2008	51		4/51 8%	4/51 8%	0/4 0%	0	6,8
Mavrogenis et al. [17]	2011	47	0/8 0%	5/39 13%	5/47 11%	1/5 25%	0	6,1
Yamamoto et al. [8]	2012	40	0/34 0%	0/6 0%	0/40 0%	0/0 0%	0	
Fisher et al. [32]	2013	63	59	4	14/63 22%	0/14 0%	0	
Mussi et al. [33]	2014	171	5/95 5%	11/76 14%	16/171 9%	0/16 0%	0	
Cassier et al. [26]	2014	283^a^	158	121	26/283 9%	0/26 0%	0	
Kito et al. [29]	2015	41	7/11 64%	0/30 0%	7/41 17%	1/7 14%	0	7,9
Chang et al. [7]	2016	45	1/11 9%	7/34 21%	8/45 18%	0/8 0%	0	5,3
*Current study*		*40*	*0/8 0%*	*3/32 9%*	3/40 8%	0/3 0%	*0*	3,1
Total		1143	15/213 7% 430	83/488 17% 613	174/1143 15%	14/174 8%	0	5,5

^a^Including patients with unknown margin status

## Discussion

The classification of well-differentiated lipomatous tumours of the extremity and the trunk wall was clarified in the last edition of the WHO manual in 2013 [3]. The term “Atypical Lipoma” is well defined and accepted. Still, controversy exists regarding the rate of local recurrence, dedifferentiation, metastatic disease, surgical margins and adequate follow-up time as well as treatment regimen. Well-differentiated liposarcomas account for approximately 50% of all liposarcomas and are hence seen relatively often [8]. The long duration of symptoms (in this study: mean > 2 years, up to more than 20 years) indicates the low aggressiveness of the tumour. The raised average age of 62 years and the fact that nearly 75% of the patients developed the tumour in the thigh underlines the slow growth potential in large soft-tissue compartments where clinical symptoms are less noticeable. In many of our cases, MRI proved the lesion either to be an atypical lipoma or a lipoma. Thickened or nodular septa (> 2 mm), non-adipose masses within the tumour, foci of T2-weighted signal lesions, prominent contrast enhancement and size greater than 5 cm have been described as useful to differentiate both lesions from each other [9–12]. Core needle biopsy with subsequent murine double-minute 2 (MDM2) and cyclin-dependent kinase 4 (CDK4) [13–15] analysis might provide more diagnostic accuracy before surgery [16]. However, due to the fact, that both lesions require the same marginal resection in our assessment, we decided for surgery without biopsy in the radiologically typical cases. Therefore, biopsy was only occasionally performed and especially in those patients at the beginning of this series.

We report an incidence of local recurrence that is half of what has been shown in several other studies with marginal resections. The most probable explanation for this difference in our view is the median follow-up time of 40 months. Only 13 out of 40 patients had a follow-up of more than 60 months. Taking into account that local recurrence developed in most of the other studies in patients more than 60 months after surgery, it is likely that our local recurrence rate will increase over time and this represents a limitation of our study. There is data indicating that the risk of local recurrence is correlated with the time of follow-up [17].

Our data also significantly supports the observation that local recurrence is more often seen in patients who already have recurrent disease [17]. In addition, a statistically significant correlation between local recurrence and marginal or wide resection is evidenced in the literature (Tab. 1). Due to the fact, that most patients have large tumours in close proximity to major vessels or nerves, a wide resection carries a considerable risk of major functional problems and / or complications. Taking into account that dedifferentiation developed only in 14 out of 1143 patients (1.2%) and metastatic disease was not seen in any of the described series, a more aggressive management including wide resections or re-excisions after primary marginal resections seems unreasonable [16]. Also in recurrent cases with close proximity to major nerves or blood vessels, re-resection is possible without substantial morbidity [18]. There are some case reports or small series of patients indicating that dedifferentiation in local recurrence might increases the risk of metastatic disease [5, 17, 19–24].

Dedifferentiation most probably occur only in a small subregion of the tumour surrounded by well-differentiated tumour which supports the concept of surgical removal and entails a much better prognosis than with other dedifferentiated sarcomas [25]. Even recurring dedifferentiated tumours might again exhibit better differentiation [6]. Dedifferentiation is much more common in retroperitoneal (17%) or groin (28%) lesions [6]. This should be taken into account in large extremity tumours extending into the pelvis or the retroperitoneum. Weiss et al., as stated before, mentioned that dedifferentiation which is more often seen in central locations might be not site-dependent, but rather time-dependent. In those locations, the tumour might grow undetected for longer times. In contrast, the experience with large and slowly growing extremity tumours as in our and many other series might in fact prove a true site-dependency.

Radiation therapy did not affect the outcome in this small series of patients with only 4 irradiated cases (no recurrence). In general, radiation therapy is effective in reducing local recurrence in R1-resections (74%) [26, 27] but the question remains, whether adjuvant radiation is necessary if the relapse could be marginally re-resected. Radiation therapy does not affect overall survival [26]. So Cassier et al. conclude that a wait-and-see policy could be adopted for R1- and R2-resected patients provided that a potential reoperation is both feasible and reasonable [26]. However, radiotherapy should be considered especially in recurrent cases where even marginal resections might produce severe functional deficits.

Follow-up time is crucial in this entity. Some authors propose a minimum of 5 years [22, 28], which in the light of the published data with a mean time to relapse of 5.5 years appears too short. As follow-up is increased in most studies, more and more recurrences are detected. The recommended observation period is hence suggested as being 8 years by some authors [29]. There are studies which show a mean time to local relapse of 16 years [8] in later re-resected patients. Regular long-term follow-up is therefore required especially in recurrent cases and should clearly exceed 5 years. We would propose 10 years in total. Whether this is done in a biannually fashion in the first 6 years and annually later as proposed [28] or in a different scheme is controversial. Due to the very low risk of dedifferentiation, clinical observation only is also regarded as being sufficient by some authors [5]. The patient may be advised to examine him- or herself. This is underlined by data showing that in most local recurrences of soft-tissue sarcomas of the extremities, the patient notices them earlier than the investigators in routine follow-up [30].

## Conclusions

ALT represents a typical diagnosis in large deep-seated lesions of the extremities, especially in patients over 60 years of age. There are several characteristics in MRI as thickened septa (> 2 mm), non-adipose masses, foci of T2-weighted lesions and contrast enhancement differentiating them from lipomas. Marginal resection of the tumour while trying to maintain the thin capsule around the lesion and only opening the tumour if necessary for the preparation of major vital structures shows an acceptable rate of local recurrence. The risk of dedifferentiation is close to 1% and metastatic disease is exceedingly rare.

## References

[1] Evans HL, Soule EH, Winkelmann RK (1979). Atypical lipoma, atypical intramuscular lipoma, and well differentiated retroperitoneal liposarcoma: a reappraisal of 30 cases formerly classified as well differentiated liposarcoma. Cancer.

[2] Kindblom LG, Angervall L, Stener B, Wickbom I (1974). Intermuscular and intramuscular lipomas and hibernomas. A clinical, roentgenologic, histologic, and prognostic study of 46 cases. Cancer.

[3] Dei Tos AP, Pedeutour F, BJA FCDM, Hogendoorn PCW, Mertens F (2013). Atypical lipomatous tumour. WHO classification of Tumours of soft tissue and bone. Volume 4.

[4] Dei Tos AP (2000). Liposarcoma: new entities and evolving concepts. Ann Diagn Pathol.

[5] Billing V, Mertens F, Domanski HA, Rydholm A (2008). Deep-seated ordinary and atypical lipomas: histopathology, cytogenetics, clinical features, and outcome in 215 tumours of the extremity and trunk wall. J Bone Joint Surg Br Vol.

[6] Weiss SW, Rao VK (1992). Well-differentiated liposarcoma (atypical lipoma) of deep soft tissue of the extremities, retroperitoneum, and miscellaneous sites. A follow-up study of 92 cases with analysis of the incidence of “dedifferentiation”. Am J Surg Pathol.

[7] Chang DH, Ma H, Liao WC, Huang MH, Wu PS (2016). Atypical Lipomatous tumors of the extremities and trunk wall-the first case series of Chinese population with 45 cases. Ann Plast Surg.

[8] Yamamoto N, Hayashi K, Tanzawa Y, Kimura H, Takeuchi A, Igarashi K, Inatani H, Shimozaki S, Kitamura S, Tsuchiya H (2012). Treatment strategies for well-differentiated liposarcomas and therapeutic outcomes. Anticancer Res.

[9] Gaskin CM, Helms CA (2004). Lipomas, lipoma variants, and well-differentiated liposarcomas (atypical lipomas): results of MRI evaluations of 126 consecutive fatty masses. AJR Am J Roentgenol.

[10] Panzarella MJ, Naqvi AH, Cohen HE, Damron TA (2005). Predictive value of gadolinium enhancement in differentiating ALT/WD liposarcomas from benign fatty tumors. Skelet Radiol.

[11] Chung WJ, Chung HW, Shin MJ, Lee SH, Lee MH, Lee JS, Kim MJ, Lee WK (2012). MRI to differentiate benign from malignant soft-tissue tumours of the extremities: a simplified systematic imaging approach using depth, size and heterogeneity of signal intensity. Br J Radiol.

[12] Hosono M, Kobayashi H, Fujimoto R, Kotoura Y, Tsuboyama T, Matsusue Y, Nakamura T, Itoh T, Konishi J (1997). Septum-like structures in lipoma and liposarcoma: MR imaging and pathologic correlation. Skelet Radiol.

[13] Weaver J, Rao P, Goldblum JR, Joyce MJ, Turner SL, Lazar AJ, Lopez-Terada D, Tubbs RR, Rubin BP (2010). Can MDM2 analytical tests performed on core needle biopsy be relied upon to diagnose well-differentiated liposarcoma?. Mod Pathol.

[14] Kimura H, Dobashi Y, Nojima T, Nakamura H, Yamamoto N, Tsuchiya H, Ikeda H, Sawada-Kitamura S, Oyama T, Ooi A (2013). Utility of fluorescence in situ hybridization to detect MDM2 amplification in liposarcomas and their morphological mimics. Int J Clin Exp Pathol.

[15] Binh MB, Sastre-Garau X, Guillou L, de Pinieux G, Terrier P, Lagace R, Aurias A, Hostein I, Coindre JM (2005). MDM2 and CDK4 immunostainings are useful adjuncts in diagnosing well-differentiated and dedifferentiated liposarcoma subtypes: a comparative analysis of 559 soft tissue neoplasms with genetic data. Am J Surg Pathol.

[16] Errani C, Cocchi S, Ali N, Chehrassan M, Righi A, Gambarotti M, Mavrogenis AF, Vanel D, Donati D (2016). Recurrence after marginal excision for atypical Lipomatous tumors versus lipomas of the extremities. Orthopedics.

[17] Mavrogenis AF, Lesensky J, Romagnoli C, Alberghini M, Letson GD, Ruggieri P (2011). Atypical lipomatous tumors/well-differentiated liposarcomas: clinical outcome of 67 patients. Orthopedics.

[18] Kubo T, Sugita T, Shimose S, Arihiro K, Ochi M (2006). Conservative surgery for well-differentiated liposarcomas of the extremities adjacent to major neurovascular structures. Surg Oncol.

[19] Laurino L, Furlanetto A, Orvieto E, Dei Tos AP (2001). Well-differentiated liposarcoma (atypical lipomatous tumors). Semin Diagn Pathol.

[20] Azumi N, Curtis J, Kempson RL, Hendrickson MR (1987). Atypical and malignant neoplasms showing lipomatous differentiation. A study of 111 cases. Am J Surg Pathol.

[21] Evans HL (2007). Atypical lipomatous tumor, its variants, and its combined forms: a study of 61 cases, with a minimum follow-up of 10 years. Am J Surg Pathol.

[22] Sommerville SM, Patton JT, Luscombe JC, Mangham DC, Grimer RJ (2005). Clinical outcomes of deep atypical lipomas (well-differentiated lipoma-like liposarcomas) of the extremities. ANZ J Surg.

[23] Bassett MD, Schuetze SM, Disteche C, Norwood TH, Swisshelm K, Chen X, Bruckner J, Conrad EU, Rubin BP (2005). Deep-seated, well differentiated lipomatous tumors of the chest wall and extremities: the role of cytogenetics in classification and prognostication. Cancer.

[24] Kooby DA, Antonescu CR, Brennan MF, Singer S (2004). Atypical lipomatous tumor/well-differentiated liposarcoma of the extremity and trunk wall: importance of histological subtype with treatment recommendations. Ann Surg Oncol.

[25] Lucas DR, Nascimento AG, Sanjay BK, Rock MG (1994). Well-differentiated liposarcoma. The Mayo Clinic experience with 58 cases. Am J Clin Pathol.

[26] Cassier PA, Kantor G, Bonvalot S, Lavergne E, Stoeckle E, Le Pechoux C, Meeus P, Sunyach MP, Vaz G, Coindre JM (2014). Adjuvant radiotherapy for extremity and trunk wall atypical lipomatous tumor/well-differentiated LPS (ALT/WD-LPS): a French sarcoma group (GSF-GETO) study. Annals Oncol.

[27] Kang J, Botros M, Goldberg S, Giraud C, Nielsen GP, Chen YL, Raskin K, Schwab J, Yoon SS, Hornicek FJ (2013). The use of radiation therapy in the management of selected patients with atypical lipomas. Sarcoma.

[28] Rozental TD, Khoury LD, Donthineni-Rao R, Lackman RD (2002). Atypical lipomatous masses of the extremities: outcome of surgical treatment. Clin Orthop Relat Res.

[29] Kito M, Yoshimura Y, Isobe K, Aoki K, Momose T, Suzuki S, Tanaka A, Sano K, Akahane T, Kato H (2015). Clinical outcome of deep-seated atypical lipomatous tumor of the extremities with median-term follow-up study. Eur J Surg Oncol.

[30] Cool P, Grimer R, Rees R (2005). Surveillance in patients with sarcoma of the extremities. Eur J Surg Oncol.

[31] Zagars GK, Goswitz MS, Pollack A (1996). Liposarcoma: outcome and prognostic factors following conservation surgery and radiation therapy. Int J Radiat Oncol Biol Phys.

[32] Fisher SB, Baxter KJ, Staley CA, Fisher KE, Monson DK, Murray DR, Oskouei SV, Weiss SW, Kooby DA, Maithel SK (2013). The general Surgeon's quandary: atypical lipomatous tumor vs lipoma, who needs a surgical oncologist?. J Am Coll Surg.

[33] Mussi CE, Daolio P, Cimino M, Giardina F, De Sanctis R, Morenghi E, Parafioriti A, Bartoli MS, Bastoni S, Cozzaglio L (2014). Atypical lipomatous tumors: should they be treated like other sarcoma or not? Surgical consideration from a bi-institutional experience. Ann Surg Oncol.

